# Peripheral platelet/lymphocyte ratio predicts lymph node metastasis and acts as a superior prognostic factor for cervical cancer when combined with neutrophil: Lymphocyte

**DOI:** 10.1097/MD.0000000000004381

**Published:** 2016-08-12

**Authors:** Liang Chen, Fang Zhang, Xiu-gui Sheng, Shi-qian Zhang, Yue-ting Chen, Bo-wen Liu

**Affiliations:** a Department of Gynecological Oncology, Shandong Cancer Hospital Affiliated to Shandong University, Shandong Academy of Medical Sciences; b Department of Radiology, Provincial Hospital Affiliated to Shandong University; c Department of Obstetrics and Gynecology, Qilu Hospital of Shandong University, Jinan, PR China.

**Keywords:** cervical cancer, lymph node metastasis, neutrophil/lymphocyte ratio, platelet/lymphocyte ratio, prognosis, sensitivity and specificity

## Abstract

Inflammation-based indicators such as neutrophil/lymphocyte ratio (NLR), derived NLR (dNLR), and platelet/lymphocyte ratio (PLR) have been reported to possess significant predictive value for several types of cancer. We investigated the predictive value of these 3 biomarkers on lymph node metastasis (LNM) and clinical outcome in patients with stage Ib1–IIa cervical cancer undergoing radical surgery.

A total of 407 patients with FIGO stage Ib1–IIa cervical cancer, who underwent radical surgery between January 2006 and December 2009 at the Department of Gynecological and Oncology of Shandong Cancer Hospital Affiliated to Shandong University were recruited. Binary logistic regression analysis was performed to determine the relationship between PLR, NLR, dNLR, and LNM. Multivariate Cox regression analysis was performed to determine the association between the 3 indices and recurrence-free survival (RFS) and overall survival (OS).

Optimal cut-off values for the 3 indices were determined by applying receiver operating curve (ROC) analysis. Univariate and binary logistic regression analyses both indicate that PLR was significantly associated with increased LNM (*P* < 0.05). In the multivariate survival analysis, increased preoperative PLR and NLR were significantly associated with reduced RFS (*P* = 0.001 and *P* = 0.002, respectively), whereas a combination of both PLR and NLR revealed a more significant association with reduced RFS (*P* < 0.001). Furthermore, increased preoperative PLR and NLR were significantly associated with reduced OS (*P* = 0.007 and *P* = 0.009, respectively), whereas the combined use of PLR and NLR revealed a more significant association with reduced OS (*P* = 0.003).

PLR is an independent risk factor for increased LNM and clinical outcome in patients with stage Ib1–IIa cervical cancer. A combination of PLR and NLR may enable better risk stratification for predicting survival.

## 1 Introduction

Recent studies have shown that both inflammatory reaction and immune status are prognostic factors of tumor formation.[ ^1^ ^2^] Tumor-associated inflammatory responses consist of inflammatory cells and a series of inflammatory mediators. Together, these generate a tumor-related inflammatory microenvironment and play vital roles in the pathogenesis and progression of tumors. ^[2]^ Furthermore, such factors may contribute to reduced sensitivity to antitumor therapy. On the other hand, tumor-induced inflammatory responses can lead to changes in hematological components such as neutrophils, lymphocytes, monocytes, and platelets.[ ^3^ ^4^] Neutrophil/lymphocyte ratio (NLR), derived NLR (dNLR), and platelet/lymphocyte ratio (PLR) are all useful inflammation-based prognostic indicators. These markers can reflect relative changes in different components of the blood, which have been proven as potential prognostic markers in various cancers. [^5^ ^6^ ^7^ ^8^ ^9^]

Cervical cancer is the third most common cancer in females worldwide and represents the fourth leading cause of cancer-related death. ^[10]^ The primary treatment of early stage cervical cancer (International Federation of Gynecology and Obstetrics [FIGO] stage IA2–IIA) is either surgery or radiation therapy (RT). Bilateral pelvic lymph node dissection, with or without para-aortic lymph node sampling, is a necessary component of primary surgical treatment. Nowadays, the prediction of cervical cancer progression or recurrence is mainly limited to the use of postoperative prognostic factors. Predictive biomarkers may enable a far better risk stratification for recurrence, in order to select appropriate treatment and provide individual adjuvant therapy following surgery. Consequently, there is significant interest in developing new prognostic biomarkers, particularly inflammatory factors, which are relatively inexpensive and readily available.

In patients with cervical cancer, NLR has been reported to act as a valuable tool in predicting therapeutic response to radiation therapy (RT) and concurrent chemoradiation therapy (CCRT). ^[3]^ Lee et al ^[11]^ further found that pretreatment NLR in patients with cervical cancer acts as a cost-effective biomarker in stratifying the risk of recurrence and death. Zhang et al ^[12]^ reported that NLR, in preference to PLR, predicts clinical outcome in cervical cancer patients treated with radical surgery. Pretreatment thrombocytosis at the time of initial diagnosis was also found to be an independent prognostic factor for cervical cancer patients treated with definitive radiotherapy. ^[13]^ In the present study, the prognostic values of 3 inflammatory factors (NLR, PLR, and dNLR) on clinical outcome in stage Ib1–IIa cervical cancer patients undergoing radical operation were systematically compared. For the first time, the effects of these 3 indices were also evaluated upon lymph node metastasis (LNM). Finally, the combined use of these ratios for the improvement of patient risk stratification was investigated.

## 2 Methods

### 2.1 Ethics statements

This study was approved by the Ethics Committee of Shandong Cancer Hospital Affiliated to Shandong University. All the procedures were performed in accordance with the Declaration of Helsinki 2013 Edition. All patients provided informed written consent prior to participating into our study.

### 2.2 Patients

Patients with FIGO stage Ib1–IIa cervical cancer, who underwent radical surgery between January 2006 and December 2009 at the Department of Gynecological and Oncology of Shandong Cancer Hospital affiliated to Shandong University, were recruited in the retrospective study. All patients were clinically staged according to the FIGO clinical staging system. Other variables in the study included age, tumor histological type, tumor grade, LNM, and lymphovascular space invasion (LVSI). All data were retrieved from patient medical records. Inclusion criteria were as follows: (1) FIGO stage Ib1–IIa. (2) Primary surgery included radical hysterectomy and bilateral pelvic lymph node dissection with (or without) para-aortic lymph node sampling. Para-aortic node dissection was performed in patients with suspected or known nodal disease. (3) Routine blood tests including neutrophil, lymphocyte, and platelet count were obtained within 2 days after admission, before commencing treatment. (4) Postoperative adjuvant therapy was performed depending on tumor stage and postoperative pathology, and according to the FIGO Guidelines for cervical cancer. Patients with hematological disease (n = 4), inflammatory disease (n = 2), missing blood cell counts (n = 2), or where follow-up data was unavailable (n = 3) were excluded. Together, 11 patients were excluded and 407 patients remained for further analyses.

### 2.3 Follow-up evaluation

Follow-up examinations were carried out at 3-month intervals within the first 2 years, at 6-month intervals for 3 to 5 years, and annually thereafter until December 2014. Routine examinations included physical and gynecological checkup, laboratory tests, and imaging methods.

### 2.4 Statistical analyses

One of the research objectives was LNM. The primary end point of the study was recurrence-free survival (RFS), which was defined as the interval between the date of diagnosis and the date of the first event (i.e., tumor recurrence or last follow-up). The second end point was overall survival (OS), which was measured from the date of diagnosis to the date of death from any cause. Optimal cut-off values for inflammatory indices were determined by applying receiver operating curve (ROC) analysis. The biggest Youden index (sensitivity+specificity-1) was selected as the optimal cut-off point. Spearman rank correlation was used to investigate correlations between these 3 indices (PLR, NLR, and dNLR). Clinical end points were measured by the Kaplan–Meier method and compared by log-rank tests. Binary logistic regression analysis was performed to determine the relationship between PLR, NLR, dNLR, and LNM. Univariate survival analysis and multivariate Cox-regression analysis were performed to determine the influence of clinicopathological features on RFS and OS. Predictive factors, which were significantly associated with end points in the univariate analysis, were included in the multivariate analysis in a forward stepwise manner. Odds ratios (ORs) or hazard ratios (HRs) were reported as relative risks with corresponding 95% confidence intervals (CIs). All statistical analyses were performed using Statistical Package for Social Sciences version 13.0 (SPSS Inc., Chicago, IL). A 2-sided *P* < 0.05 was considered statistically significant.

## 3 Results

### 3.1 Patient characteristics and ROC curves for LNM, RFS, and OS

Median age at the time of diagnosis was 44 years, whereas median values for PLR, NLR, and dNLR were 144.62, 2.41, and 1.73, respectively. Clinicopathological characteristics of patients are shown in Table 1. Among the 407 patients, 85 (20.9%) patients developed LNM. As LNM is an important prognostic factor in predicting clinical outcome, it was analyzed separately from other data.

**Table 1:**
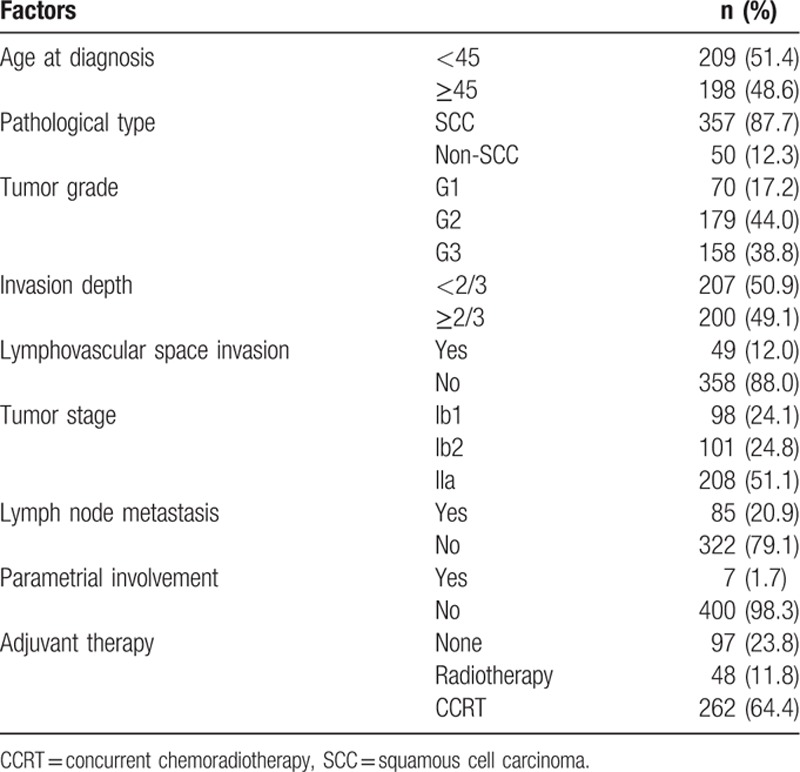
Baseline patient characteristics.

**Figure 1:**
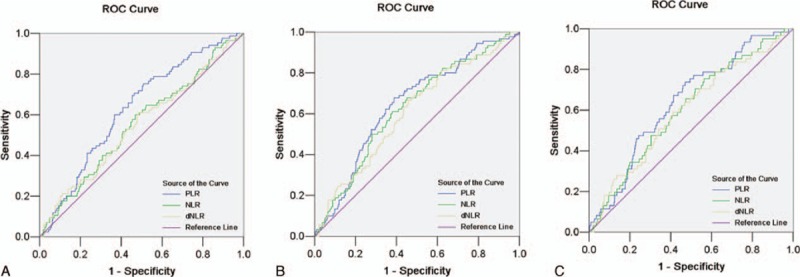
For PLR, NLR, and dNLR, the areas under the curve were 0.630, 0.553, and 0.547, respectively, for LNM (A), 0.645, 0.626, and 0.608, respectively, for RFS (B), and 0.641, 0.606, and 0.598, respectively, for OS (C). NLR = neutrophil/lymphocyte ratio, dNLR = derived NLR, PLR = platelet/lymphocyte ratio, ROC = receiver operating characteristic, LNM = lymph node metastasis, RFS = recurrence-free survival, OS = overall survival.

ROC curves were performed to determine the cut-off points of PLR, NLR, and dNLR for LNM, RFS, and OS. By applying ROC analysis, optimal cut-off levels for PLR, NLR, and dNLR were determined as 138.35, 2.42, and 1.68 for LNM, 152.02, 2.59, and 1.71 for RFS, and 143.47, 2.09, and 1.85 for OS, respectively. Areas under the curves for PLR, NLR, and dNLR were 0.630, 0.553, and 0.547, respectively, for LNM, 0.645, 0.626, and 0.608, respectively, for RFS, and 0.641, 0.606, and 0.598, respectively, for OS. ROC curves for LNM, RFS, and OS are shown in Fig. 1A–C.

When stratified by cut-offs for RFS and applied to nonparametric tests, NLR was significantly associated with parametrial involvement (Fisher's exact test, *P* = 0.022), whereas PLR was significantly associated with age, tumor-invasion depth, and LVSI (all *P* < 0.05). When stratified by cut-offs for OS, PLR was also significantly associated with age, tumor-invasion depth, and LVSI (all *P* < 0.05). However, no clinicopathological parameters were revealed to be significantly associated with NLR (all *P* > 0.05).

### 3.2 Binary logistic regression analysis for LNM

When patients were stratified by cutoffs for LNM, our study revealed that all inflammatory indices, tumor-invasion depth, and LVSI were significantly associated with LNM when analyzed by univariate analysis (all *P* < 0.05, Table 2). These factors were included in the binary logistic regression analysis in a forward stepwise manner. By applying Spearman's rank correlation coefficient, PLR, NLR, and dNLR were significantly correlated with each other (Spearman's rho coefficients were 0.454 [NLR vs PLR], 0.401 [PLR vs dNLR], and 0.902 [NLR vs dNLR), all *P* < 0.001). Consequently, these 3 inflammatory indices were included in the binary logistic regression analysis. The analysis revealed that there was a significant association between inflammatory indices, tumor-invasion depth, and LVSI with LNM (all *P* < 0.05, Table 2). By applying ROC curves and binary logistic regression analyses, it was determined that PLR was superior for predicting LNM in cases of cervical cancer.

**Table 2:**
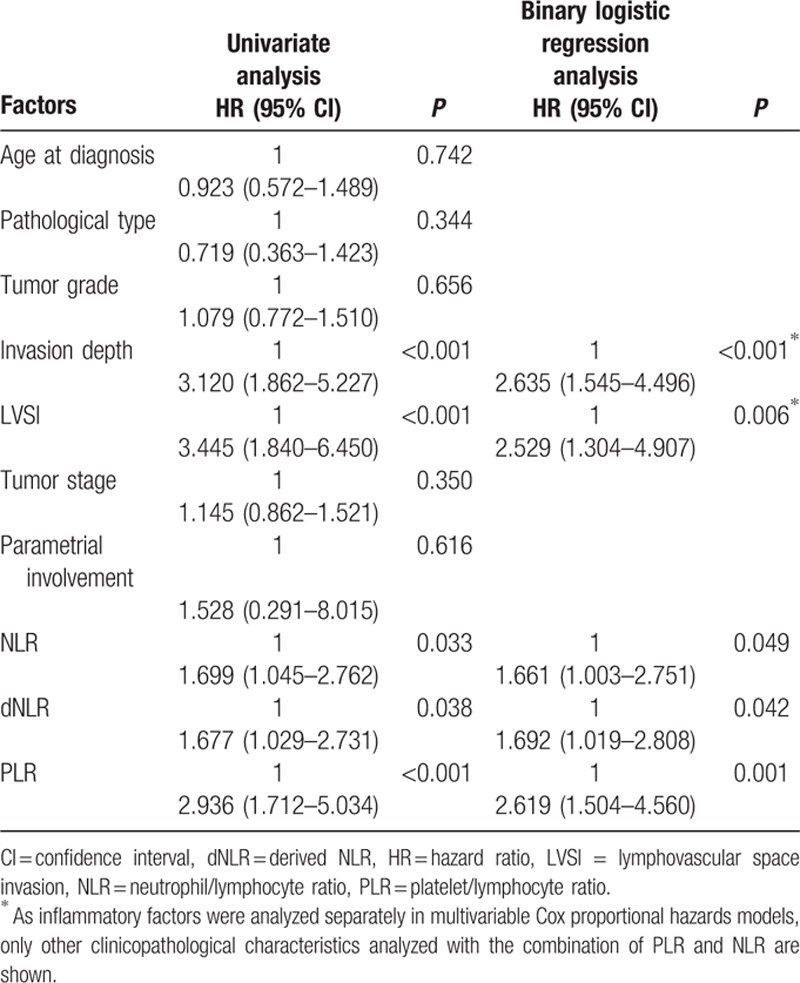
Univariate and binary logistic regression analysis of the association between prognostic factors and lymph node metastasis.

### 3.3 Univariate survival analysis and multivariate Cox-regression analysis for patients stratified according to PLR, NLR, and dNLR cut-offs and other clinicopathological characteristics

**Figure 2:**
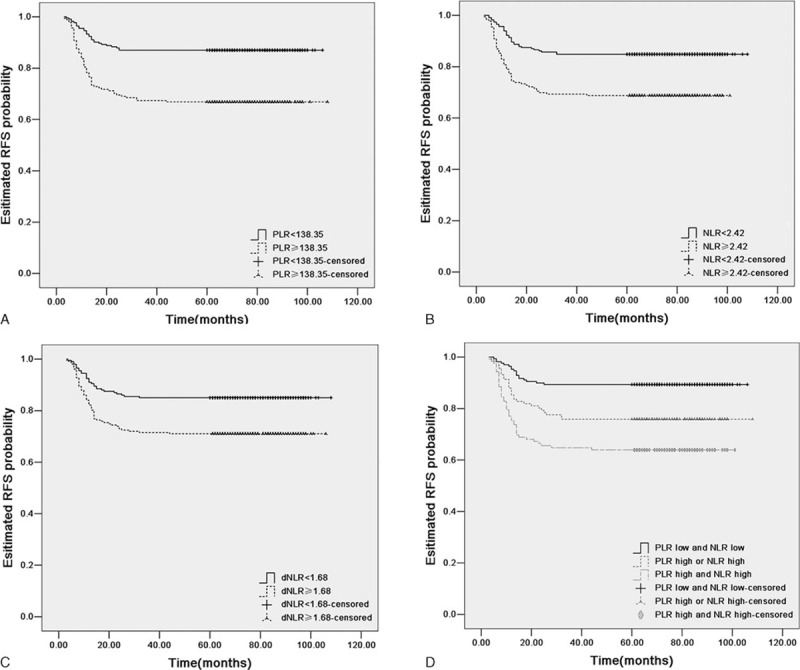
Kaplan–Meier RFS curves plus log-rank *P*-values for patients stratified using PLR cut-off (A, *P* *<* 0.001), NLR cut-off (B, *P* *<* 0.001), dNLR cut-off (C, *P* = 0.001), and the combination of PLR and NLR (D, *P* *<* 0.001). NLR = neutrophil/lymphocyte ratio, dNLR = derived NLR, PLR = platelet/lymphocyte ratio, RFS = recurrence-free survival.

**Figure 3:**
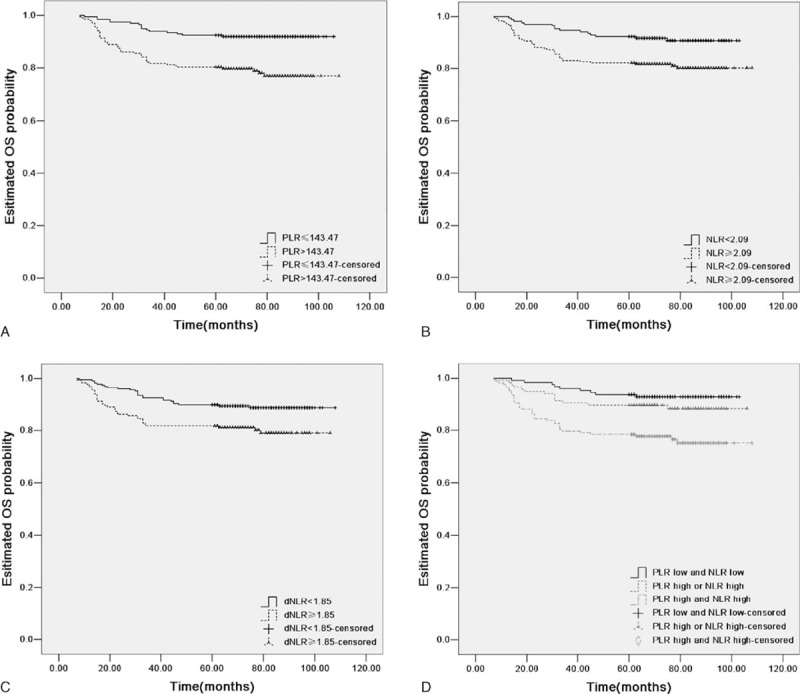
Kaplan–Meier OS curves plus log-rank *P*-values for patients stratified using PLR cut-off (A, *P* *<* 0.001), NLR cut-off (B, *P* = 0.004), dNLR cut-off (C, *P* = 0.009), and the combination of PLR and NLR (D, *P* *<* 0.001). NLR = neutrophil/lymphocyte ratio, dNLR = derived NLR, PLR = platelet/lymphocyte ratio, OS = overall survival.

For PLR, NLR, and dNLR, cut-off levels for RFS or OS were selected as the uniform point for a series of survival analyses. Univariate analysis revealed that there was a significant association between tumor-invasion depth, LVSI, parametrial involvement, LNM, tumor stage, and adjuvant therapy with RFS and OS (all *P* < 0.05, Tables 3 and 4). PLR, NLR, and dNLR were all significant prognostic factors for RFS (all *P* < 0.05; Table 3; Fig. 2A–C) and OS (all *P* < 0.05; Table 4; Fig. 3A–C).

**Table 3:**
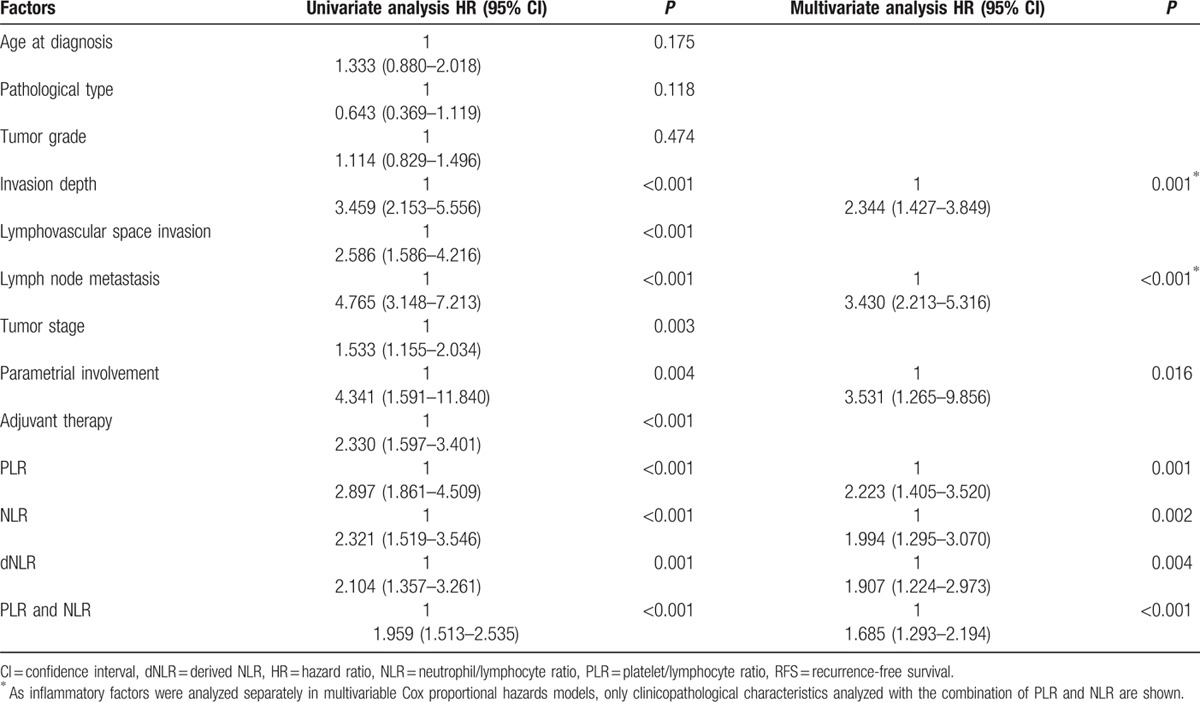
Recurrence-free survival (RFS) of cervical cancer patients stratified according to PLR cut-offs, NLR cut-offs, and a combination of PLR and NLR cut-offs, together with other prognostic factors.

**Table 4:**
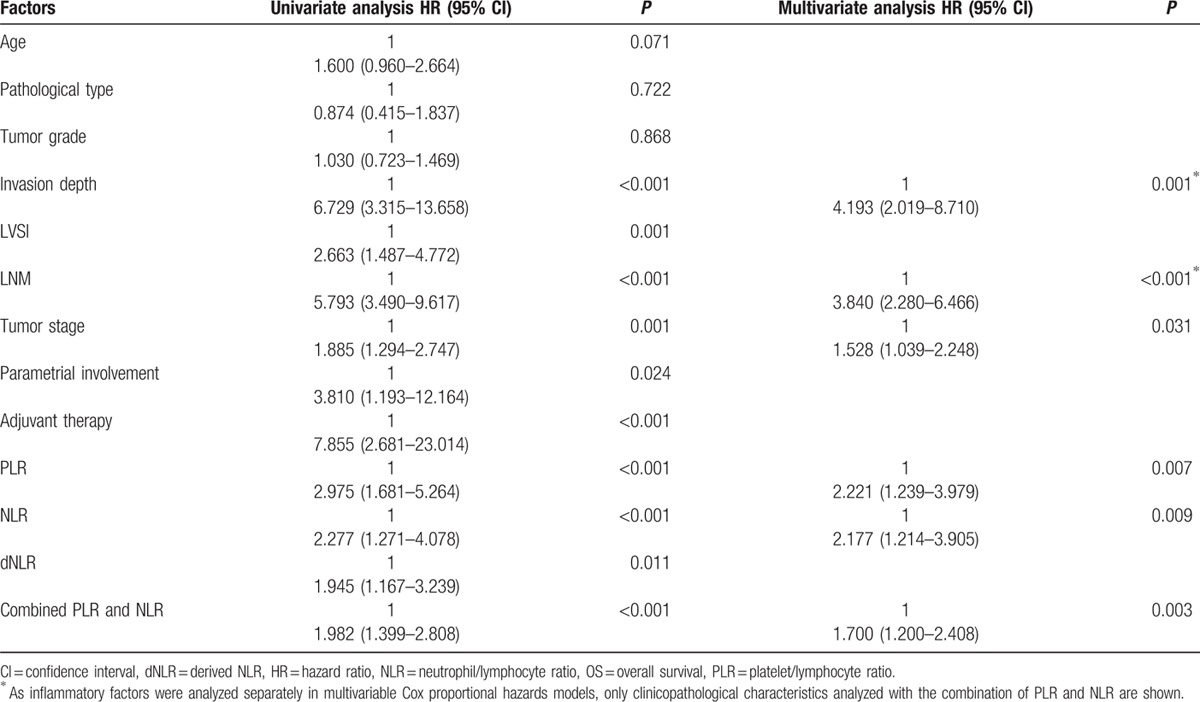
Overall survival (OS) of cervical cancer patients stratified according to PLR cut-offs, NLR cut-offs, and a combination of PLR and NLR cut-offs, together with other prognostic factors.

Prognostic factors that were significantly associated with end points in the univariate analysis were included in the multivariate Cox-regression analysis in a forward stepwise manner. By applying Spearman's rank correlation coefficient, PLR, NLR, and dNLR were significantly correlated with each other (all *P* < 0.001). Consequently, all 3 factors were analyzed separately in multivariable Cox proportional hazards models. For RFS, these 3 factors all remained as independent prognostic factors (Table 3; all *P* < 0.05). For OS, PLR and NLR remained significant in the multivariate analysis (Table 4, all *P* < 0.05), whereas dNLR did not represent an independent prognostic factor.

### 3.4 Combining PLR and NLR ratios to improve patient risk stratification

Methods were investigated to combine PLR and NLR in an attempt to improve patient risk stratification in relation to clinical outcome. Combining NLR and PLR did not provide any further prognostic value, compared with PLR or NLR individually. For RFS, we found that a simple and effective approach was to stratify patients into 3 groups according to PLR and NLR cut-offs: (1) PLR-low and NLR-low, (2) PLR-high or NLR-high, and (3) PLR-high and NLR-high (baseline patient characteristics stratified by the combination of PLR and NLR for RFS, Table 3). These corresponded to low, intermediate, and high-risk groups with estimated 5-year RFS rates of 89.3%, 75.9%, and 63.9%, respectively. There were significant statistical differences when compared between any 2 groups (log-rank test, all *P* < 0.005; Fig. 2D).

According to PLR and NLR cut-offs for OS, we stratified patients into a similar group with estimated 5-year OS rates of 92.8%, 88.7%, and 76.6%, respectively. Significant differences were observed between any 2 groups (log-rank test, all *P* < 0.005; Fig. 3D), except for the low and intermediate risk groups (log-rank test, *P* = 0.256).

In the univariate analysis, there were no accentuated differences among PLR, NLR, or a combination of the 2 indices. In the multivariate analysis, increased preoperative PLR and NLR were significantly associated with reduced RFS (*P* = 0.001 and *P* = 0.002), respectively, whereas a combination of PLR and NLR was associated with a more significant reduction in RFS (*P* < 0.001). Furthermore, increased preoperative PLR and NLR were significantly associated with reduced OS (*P* = 0.007 and *P* = 0.009, respectively), whereas a combination of these 2 indices were associated with a more significant reduction in OS (*P* = 0.003).

For further exploration of the optimal cut-offs for combining PLR and NLR in clinical outcome, the median value of PLR and NLR were chosen as cut-off points. The median values of PLR and NLR were 144.62 and 2.41, respectively. In univariate analysis, elevated PLR, NLR, and a combination of these indices were significantly associated with reduced RFS. In multivariate analysis, an increased preoperative PLR or NLR were significantly associated with reduced RFS (*P* = 0.003 and *P* = 0.002, respectively), whereas a combination of these indices was associated with a more significant reduction in RFS (*P* < 0.001). Increased preoperative PLR and NLR were significantly associated with reduced OS (*P* = 0.007 and *P* = 0.030, respectively), whereas a combination of these indices was associated with significantly reduced RFS (*P* = 0.004).

## 4 Discussion

Pretreatment peripheral inflammatory cells have been shown to be readily available and trusted markers that reflect systemic inflammatory response to the manifestations of cancer. Lymphocytes act as crucial components of host immunity, and reductions in such cells lead to disorders of tumor immunity. [^14^ ^15^ ^16^] Reductions in tumor-infiltrating CD4+ T cells and reversed CD4/CD8 ratios have been found to be significantly related to rapid tumor growth and LNM in cervical cancer. ^[17]^ In contrast, high platelet hyperlipidemia has been reported in various types of cancer and is considered to be an important prognostic indicator for many tumors including cervical cancer.[ ^13^ ^18^ ^19^] Elevated neutrophil levels were also reported to represent a useful prognostic factor for the recurrence of cervical cancer. ^[20]^ Thus, PLR, NLR, and dNLR represent independent prognostic factors for survival in several cancers. A series of 3 studies all determined that pretreatment NLR acts as a prognostic factor in patients with cervical cancer treated with initial radical surgery or radiation.[ ^3^ ^11^ ^12^] Our present study confirms previous findings, in which NLR represents an independent prognostic marker for recurrence and OS in cervical cancer.

For young patients, LNM is one of the contraindications for fertility-sparing surgery.[ ^21^ ^22^] For patients who do not desire fertility preservation, the usual suggestion from some experts is to abandon hysterectomy and provide postoperative chemoradiation if positive lymph nodes are found during surgery. ^[23]^ For patients with advanced tumors (FIGO stage IB2 to IVA stage IIb to IV, and partially stage IIa2 and Ib2), primary chemoradiation is suggested, and the volume of RT is guided by assessing the involvement of pelvic and para-aortic nodes. ^[23]^ Consequently, when considering primary treatment options for cervical cancer, physicians should note whether LNM is apparent or not. Nowadays, the detection of positive para-aortic and pelvic lymph nodes is mainly limited to imaging examinations, lymph node dissection or sentinel lymph node (SLN) mapping.[ ^24^ ^25^] There is an urgent need for predictive biomarkers for LNM, which may enable better risk stratification in order to select the appropriate treatment option for patients. Our study confirms previous findings for cervical cancer, in which factors including tumor-invasion depth and LVSI are associated with LNM. Furthermore, for the first time, we have revealed that PLR acts as a good biomarker in predicting LNM by applying ROC curves and binary logistic regression analysis. In cervical cancer patients who receive radical surgery, a high PLR grade is indicative of a higher risk of LNM and suggests the need for para-aortic lymph node biopsy. In advanced cervical cancer patients, PLR classification can assist imaging studies in the assessment of LNM, which may minimize the need for pelvic lymphadenectomy, and could help in determining the appropriate range of radiation.

For the first time, we also found that PLR acts as an independent prognostic factor for RFS and OS. In contrast to the findings of Zhang et al, ^[12]^ our study revealed that PLR has a distinct advantage over NLR in predicting the clinical outcome of cervical cancer. In a manner that is different from other post-operative risk factors, PLR acts as a preoperative index in predicting LNM and may provide useful information for determining surgical and postoperative treatments.

In multivariate analysis, dNLR was significantly associated with RFS, but not with OS. By applying ROC curves and multivariate Cox proportion analyses, our study revealed that dNLR possesses a similar prognostic value as NLR in predicting the clinical outcome of cervical cancer. However, our data also revealed that NLR exhibits a small superiority in the prognostic value over dNLR, as reported for other types of cancers.[ ^6^ ^26^] Thus, only NLR and PLR were included in our investigation of combined ratios in an attempt to improve patient risk stratification in relation to clinical outcome. By combining NLR and PLR scores clarified by cut-offs for RFS or OS, we attempted to stratify patients into low (PLR-low, NLR-low), intermediate (PLR-high or NLR-low), and high (PLR-high, NLR-high) risk groups. There were statistically significant differences when comparing between any 2 groups clarified according to cut-offs for RFS (log-rank test, all *P* < 0.005; Fig. 2D). Statistically significant differences were found after applying the log-rank test when comparing between any 2 groups, except for low and intermediate risk groups in OS (Fig. 3D). Thus, NLR and PLR could be combined to provide additional risk stratification for cervical cancer patients, as both indices represent biomarkers of cancer-related inflammation, which only partially overlaps with regard to prognostic information. PLR or NLR can also act as preoperative indices in predicting clinical outcome and may provide useful information for selecting appropriate options for surgery and postoperative adjuvant therapy. A combination of these 2 indices provides better risk stratification to select appropriate treatment options for patients.

In this study, we exhibited that the optimal cut-off point for PLR or NLR represents a preliminary factor for distinguishing a prognostic level. However, it is not possible to completely exclude the potential for selection bias from this study. First, all patients were all diagnosed by cervical biopsy. It is possible that cervical biopsy may have exerted influence over local inflammation. Furthermore, the time interval between biopsy and admission differed between patients. In addition, this study was designed as a retrospective investigation, and such designs are known to be prone to some degree of selection bias. The strength of the current study is the large sample size and short time interval for blood sample collection (within 2 days of admission). It is well-known that either antitumor or anti-inflammatory therapy may cause changes in the immune system. The short time interval for blood sampling was important, as it excluded any possible interference from treatment or drug administration.

## 5 Conclusions

In summary, we first demonstrated that pretreatment peripheral PLR predicts LNM and clinical outcome in patients with stage Ib1–IIa cervical cancer. Furthermore, a combination of PLR and NLR enabled better risk stratification for predicting survival. As PLR can be directly derived from routine blood cell counts, it may prove to be a readily available and trusted marker for patient stratification and individual risk assessment. Thus, the optimal cut-off point for PLR or NLR represents a preliminary factor for distinguishing prognostic characteristics. Before this method can be unified and deployed in routine clinical research, a prospective study is very much required.
